# Safety and Efficacy of Methotrexate in Psoriasis: A Meta-Analysis of Published Trials

**DOI:** 10.1371/journal.pone.0153740

**Published:** 2016-05-11

**Authors:** Jonathan West, Simon Ogston, John Foerster

**Affiliations:** 1 University of Dundee, College of Medicine, Dentistry, and Nursing, Dundee, Scotland; 2 Department of Dermatology and Photobiology, NHS Tayside, Dundee, Scotland; Center for Rheumatic Diseases, INDIA

## Abstract

**Background:**

Methotrexate (MTX) has been used to treat psoriasis for over half a century. Even so, clinical data characterising its efficacy and safety are sparse.

**Objective:**

In order to enhance the available evidence, we conducted two meta-analyses, one for efficacy and one for safety outcomes, respectively, according to PRISMA checklist. (Data sources, study criteria, and study synthesis methods are detailed in Methods).

**Results:**

In terms of efficacy, only eleven studies met criteria for study design and passed a Cochrane risk of bias analysis. Based on this limited dataset, 45.2% [95% confidence interval 34.1–60.0] of patients achieve PASI75 at primary endpoint (12 or 16 weeks, respectively, n = 705 patients across all studies), compared to a calculated PASI75 of 4.4 [3.5–5.6] for placebo, yielding a relative risk of 10.2 [95% C.I. 7.1–14.7]. For safety outcomes, we extended the meta-analysis to include studies employing the same dose range of MTX for other chronic inflammatory conditions, e.g. rheumatoid arthritis, in order not to maximise capture of relevant safety data. Based on 2763 patient safety years, adverse events (AEs) were found treatment limiting in 6.9 ± 1.4% (mean ± s.e.) of patients treated for six months, with an adverse effect profile largely in line with that encountered in clinical practice. Finally, in order to facilitate prospective clinical audit and to help generate long-term treatment outcomes under real world conditions, we also developed an easy to use documentation form to be completed by patients without requirement for additional staff time.

**Limitations:**

Meta-analyses for efficacy and safety, respectively, employed non-identical selection criteria.

**Conclusions:**

These meta-analyses summarise currently available evidence on MTX in psoriasis and should be of use to gauge whether local results broadly fall within outcomes.

## Introduction

### Rationale

Although used for decades to treat psoriasis, surprisingly little systematic review of the use of methotrexate (MTX) in psoriasis has been carried out. A recent meta-analysis, comparing several systemic psoriasis treatments, did include methotrexate [1]. It is instructive that these authors identified approx. 16,000 patients on biologic drugs versus approx. 1800 on conventional systemic drugs, likely reflecting an evidence bias favouring more expensive drugs being subjected to high quality clinical studies. This study reported comparative efficacy of methotrexate, mostly to biologics, while no attempt was made to derive an estimate of PASI75 results versus placebo for methotrexate across clinical trials.

In MTX treatment, safety considerations are often limiting in addition to efficacy. A major safety determinant is the MTX dose employed. If comparable dose ranges are employed, safety outcomes reported in other common chronic inflammatory conditions will thus be informative for psoriasis, even if the pathogenesis of the underlying condition treated differs. We therefore performed a second, extended, meta-analysis to include not only studies on psoriasis but also psoriatic arthritis, rheumatoid arthritis, Crohn’s disease, palmoplantar psoriasis, as well as sero-negative spondyl-arthropathy. Although the co-morbidity spectrum of these patient populations will be not identical to psoriasis patients, including studies on these related indications in a meta-analysis vastly increases the sample size relevant for safety outcomes.

Pharmaco-economic considerations are increasingly important in guiding treatment decisions. Almost universally, drugs such as MTX will be administered initially in routine practice, only to be replaced by biologics if found ineffective, poorly tolerated, or contra-indicated. Thus, in order to ensure that local practice exhausts all reasonable efforts to achieve sufficient disease control in psoriasis patients before introducing more costly treatments, it is paramount that MTX treatment be audited to establish that local outcomes in terms of safety and efficacy are in line with expected outcomes. However, this presupposes the availability of a point of reference, which currently is lacking.

### Objective

We here present meta-analyses of MTX studies, both in terms of efficacy in psoriasis, as well as safety (chronic inflammatory conditions employing same dose range) in order to update currently available evidence on MTX in psoriasis and, at the same time, provide a tentative set of criteria that hopefully will be useful for audit purposes.

## Methods

### Eligibility criteria

**Fig 1:**
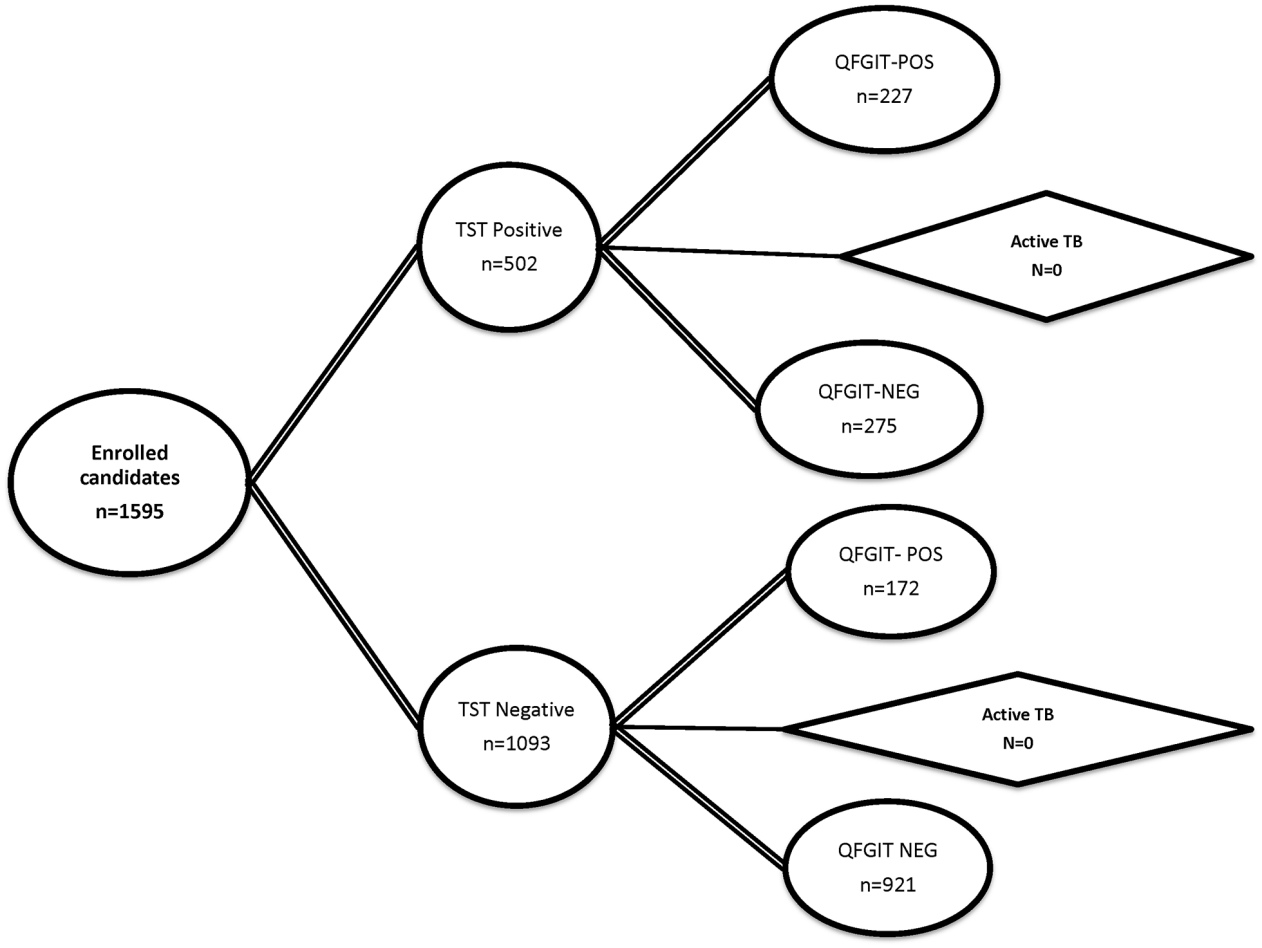
PRISMA flow diagram according to [50] summarising study selection for clinical trials reporting safety (left) and efficacy (right) outcomes, respectively. Search terms employed were: methotrexate [Title] AND (psoriasis [Title] OR arthritis [Title] OR Crohn’s [Title] OR ulcerative colitis [Title] OR ankylosing spondylitis [Title]) AND (trial [Title] OR Study [Title]) for safety studies and: Search: methotrexate [Title] AND psoriasis [Title] AND (trial [Title] OR Study [Title]) for efficacy studies.

Study selection for the meta-analysis of safety outcomes: Studies selected for safety reporting on methotrexate were identified in accordance with PRISMA guidelines as summarised in Fig 1 and detailed below (local R&D guidelines did not require assignment of a review protocol number). The PRISMA checklist is supplied as supplement (S1 File).

#### Information sources

An initial search of PubMed, EMBASE, Science Citation Index, and the Cochrane Library was performed using the SEARCH terms in the legend to Fig 1. This set was then reduced by applying the following filter: number of patients on a methotrexate only treatment arm ≥ 50, treatment duration ≥ 12 weeks, dosing oral or intramuscular (i.m.) or subcutaneously (s.c.), RCT design, as well as clearly reported adverse events. This returned 51 entries. Further full-text-based screen resulted in omission of additional 17 studies (IV dosing, non-RCT design, non-reporting of patient numbers on MTX or duration of treatment, patient number below threshold) resulting in a final set of 34 studies (references [2–35]).

Study selection for the meta-analysis of efficacy outcomes: number of patients on methotrexate-only arm ≥ 15, treatment duration ≥ 12 weeks, RCT design, and clearly reported psoriasis efficacy outcomes. Of note, the threshold for patient number per trial chosen for efficacy was lower than that for safety (n = 15 vs. n = 50) purely on pragmatic grounds as application of the more stringent criteria would have left only four studies. For efficacy studies the search terms were: psoriasis/ methotrexate/ trial, yielding an initial set of 45 entries. Filtering of abstract and title using the criteria above reduced this number to 15. The number was further reduced by two on full-text inspection as several papers reported results from the same trial, did not follow RCT design, had alternative indication (palmoplantar psoriasis), resulting in a final set of thirteen studies (references [3, 23, 25, 36–44]). Three studies (references [3, 23, 25]) fulfil the inclusion criteria for efficacy and safety analysis and are thus included in both datasets.

#### Data collection

All outcomes collected from all individual studies are detailed in S2 and S3 Files).

### Risk of Bias assessment and principle summary measures

**Fig 2:**
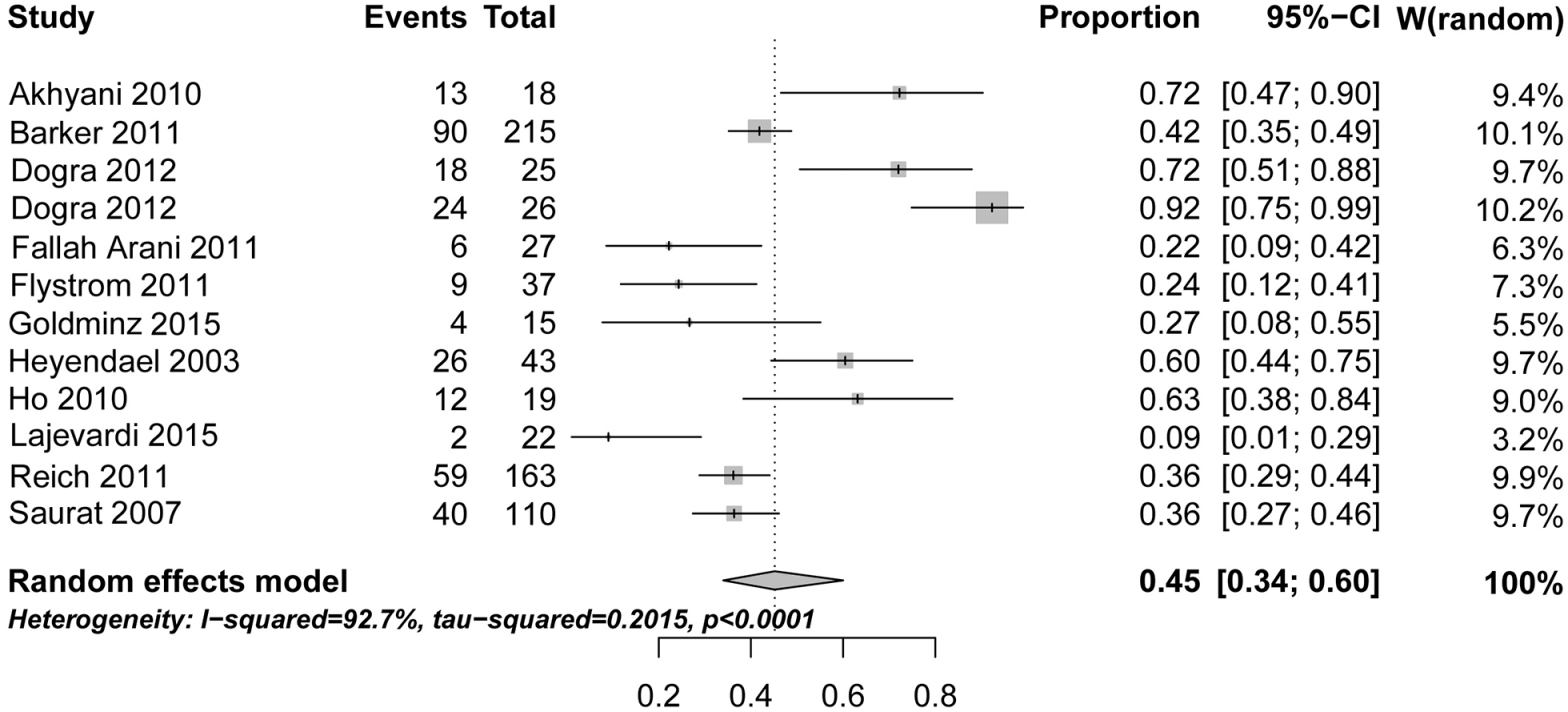
Forest plot of efficacy of MTX in psoriasis as reported in the published clinical studies shown in Table 4. The risk ratio shown refers to the PASI75 outcome reported at 12 or 16 weeks, respectively, assuming a random effects model (http://ije.oxfordjournals.org/content/39/2/421.full). One study (Dogra 2012) is listed twice because two distinct sub-cohorts were dosed differently (10 mg vs 25 mg), as described in the text.

The Cochrane Risk of Bias Assessment tool was used [45] both for the safety studies, as well as efficacy studies. For efficacy outcomes, the risk ratio between MTX and placebo was used (see Fig 2).

### Data items and synthesis of results

Spreadsheets were established to capture both efficacy and safety outcomes (S4 File, S1–S4 Tables). Incidence figures reported for the various AEs in each study were combined using a random effects model to allow for variability between studies. A random effects (Der-Simonian-Laird) meta-analysis [45] was performed on the incidence rates, yielding a pooled estimate of the mean incidence with 95% confidence interval and standard error. Handling of zeros: an incidence value of 0.5 was added in the case of a zero incidence. In addition, we also explored changing continuity correction of zeros to 0.1 from 0.5 but did not observe any notable difference in the overall results.

For the purpose of analysis AEs were classified into two groups: all AEs vs. treatment limiting AEs. Where an AE was not reported in a study, no imputation of data was made. Patient safety years were calculated for each study using the duration of methotrexate treatment and number of patients available within the studies. The total number of AEs, the total number of severe AEs, the number of AEs likely due to methotrexate treatment and also the number of deaths from each study were also captured. For meta-analysis of PASI75 outcomes in efficacy studies, a similar procedure using an Der-Simonian -Laird estimate was used to allow for calculation of mean AE rate in MTX-treated cohorts relative to patients allocated to placebo arms, followed by calculation of risk ratio between the two pooled estimates (methotrexate and placebo, respectively) as reported in Results

## Results

### Methotrexate safety studies

As detailed above, in order to maximise the available database regarding safety of Methotrexate, the meta-analysis of studies for safety outcomes reported here includes, in addition to psoriasis, indications where a shared dose is used, and with an overlapping co-morbidity and demographic spectrum, that is, psoriatic arthritis, rheumatoid arthritis, Crohn’s disease, palmoplantar psoriasis, as well as sero-negative spondyl-arthropathy. Table 1 summarises the studies analysed to obtain MTX safety outcomes. The detailed set of all AEs extracted from each study is contained in the Supplement (S1 Table). As expected, the number of patients treated for non-psoriasis conditions exceed those for psoriasis and psoriasis arthritis by almost ten-fold. As evident from the table, the dose range used for these indications is comparable to those used in psoriasis, further validating the selection of the above indications for safety analysis.

**Table 1:** Published studies reporting MTX safety outcomes relevant for psoriasis treatment^1^.

Indication	All studies(n = 34)		Psoriasis and Pso-Arthritis(n = 5)	
	Total	Range^2^	Total	Range
Nr of patients	5995	50–517	651	54–215
Patient safety years	5083	13–514	371	18–163
Treatment duration (months)	6 (Median)	3–24	5.5 (Median)	4–12
Dose (mg)	13.75 (Median)	7–25	15 (Median)	12.5–17.5

^1^ Clinical studies were identified and selected as detailed in Methods. Included were studies for psoriasis, psoriasis arthritis, rheumatoid arthritis, and Crohn’s disease. Additional studies for related conditions, e.g., ankylosing spondylitis, were screened (see S1 Table) but removed based on either failed inclusion criteria or risk Cochrane assessment.

^2^ Range of variables across individual studies.

### Methotrexate-associated adverse events (AE)

**Fig 3:**
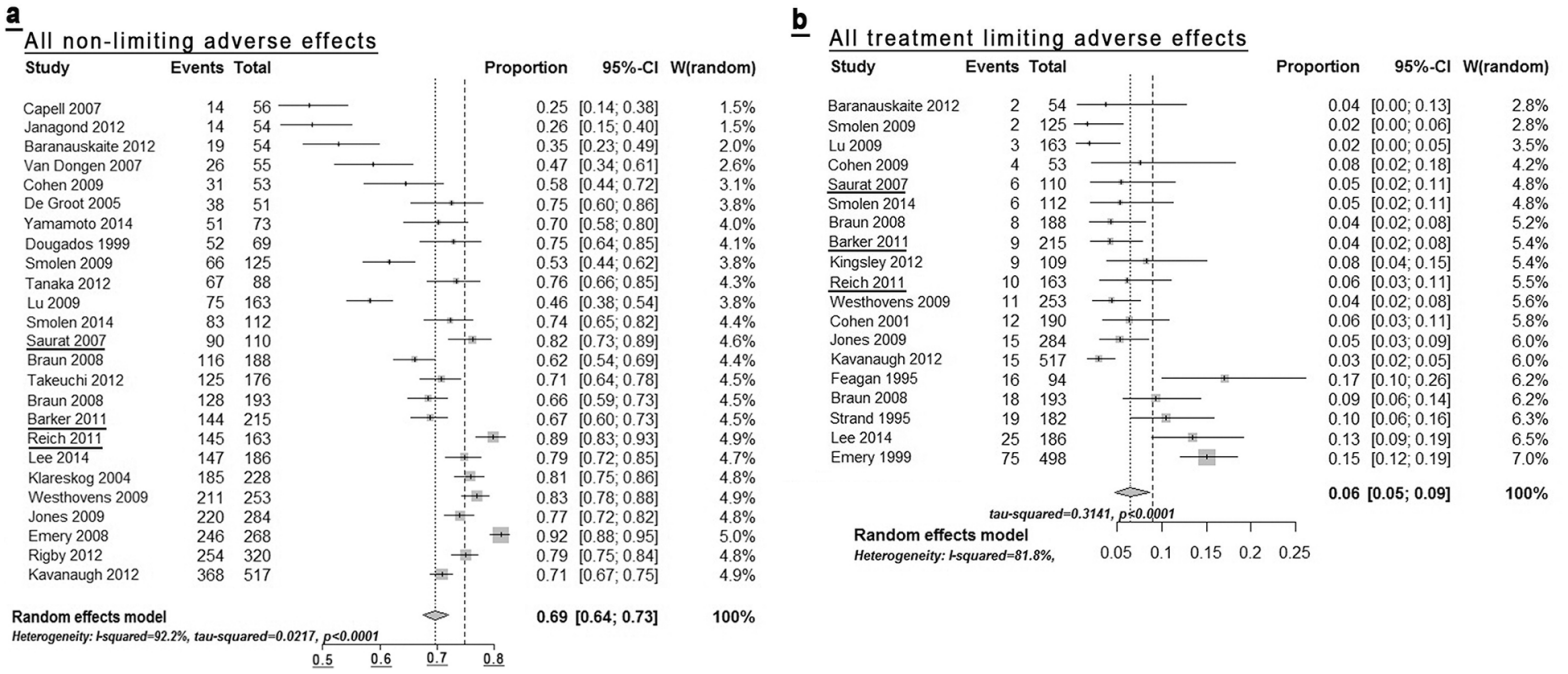
Forest plot of all non treatment limiting adverse effects (Fig3a), as reported in the studies summarised in Table 2, as well as limiting AEs (Fig 3b). All individual study data and forest plots for all individual AE’s are detailed in the Supplement. Underlined studies were conducted for psoriasis as indication.

We identified a total of 34 studies describing methotrexate administration for at least 3 months, applying the search criteria detailed in Methods. Table 2 summarises all AEs reported in at least four studies with an incidence of > 0.1%. (The results for all studies are listed in S2 Table.) An unavoidable general limitation of this meta-analysis is that AEs had to be extracted from sub—cohorts of patients exposed to MTX compared to other treatment arms, e.g. placebo and/or comparator drugs. Thus, the numbers listed do not show excess incidence attributable to MTX exposure, as compared to placebo. The Forest plot of the summary data (Fig 3a) indicates that one factor underlying the high degree of heterogeneity is an apparent under-reporting of AE’s in studies with small sample size. Indeed, closer examination of individual AEs reveals that most AE’s appear systematically less frequent in the smaller studies (S4 File). Notably, some—even rare—AE’s, are present with low heterogeneity (S5 File, highlighted in bold print in Table 2). Among these, the incidence of two AEs (pneumonia 0.8%, severe infections, 1.6%), is reported in multiple studies. The frequencies of these AEs therefore appear to be robust against sample size, protocol design, etc and may be most expected to be replicable under real world conditions. The only AE that is highly heterogenous without apparent bias due to study size is nausea/vomiting (S4 File). The reported AE spectrum as such is mostly in line with existing drug labelling information. The most obvious overall limitation in the data is that MTX exposure in published trials rarely exceeds 18 months; more commonly it is limited to 12 months. Therefore, it is impossible to extrapolate the expected incidence of any delayed onset AEs. Another limitation is that reporting of AE terms varies greatly, such that no study reports all of the safety outcomes. Nonetheless, the AE spectrum detailed in Table 2 yields a basis exceeding 1000 patient safety years for the majority of AEs.

**Table 2:** Adverse effects associated with MTX treatments in published trials^1^.

AE term^4^	Incidence^2^	Range	Studies reporting	Duration^3^(months)	Safety years^5^
All infections	27.6%	3–64.5	14	12 (3–18)	2430
N/V	18.2%	2–42.6	22	6 (3–24)	3117
**Mouth ulcers**	**11.1%**	0–14	4	12 (3–24)	792
URI	10.2%	0.6–39	13	11 (6–24)	2424
Abnormal LFTs	10.0%	1–23.9	17	6 (3–24)	1997
Abdominal pain	7.5%	1.1–18	8	6 (4–24)	1060
Headache	7.3%	0.8–27	17	6 (3–24)	2501
Alopecia	7.3%	2.7–12	5	12 (6–24)	1370
Diarrhoea	6.8%	1.2–21.6	17	6 (3–24)	2532
Sinusitis	6.6%	0.2–17	5	12 (6–24)	1471
**Cough**	**6.4%**	2.2–7.5	5	12 (3–24)	877
Fatigue	6.1%	1.8–16	9	6 (3–18)	895
Rash	6.0%	0.6–23	8	12 (4–12)	1705
Dizziness	4.7%	1–11	6	9 (3–24)	854
**Insomnia**	**4.6%**	2.1–5.7	3	4 (3–12)	221
**Leucopenia**	**3.4%**	1–5.9	5	6 (3–18)	265
UTI	2.9%	0.6–7.2	5	6 (3–11)	537
Pruritus	2.3%	0–5.6	6	9 (4–24)	1271
**Severe infection**	**1.6%**	0–4.4	14	11.5 (4–24)	2711
All malignancy	1.2%	0–2	11	12 (5.5–24)	2465
**Pneumonia**	**0.8%**	0–3.9	7	11 (4–24)	1760
AELTX^6^	28.3%	3.7–52.8	8	6 (5.5–24)	968

^1^ Data shown include adverse events reported in at least three independent studies with >200 total patient safety years and with a weighted incidence of > 0.1%. The full data set containing all reviewed studies is provided in the Supplement. Weighted average rates were calculated using the metaprop procedure in the R package 'meta' ((see http://finzi.psych.upenn.edu/library/meta/DESCRIPTION for details). A log transform was used and 0.5 is added to all cell frequencies of studies with a zero cell count.

^2^ Incidence shown is a weighted incidence to account for the variability of patient numbers across studies, as detailed in Methods.

^3^ Median duration across studies reporting a given AE.

^4^ Abbreviations: LFT—liver function tests, N/V—nausea and vomiting; URI—upper respiratory infection; UTI—urinary tract infection.

^5^ Total patient safety years underlying each AE. The average number of safety years per study is contained the Supplement (S1 Table).

^6^ AELTX—all adverse events judged as likely due to treatment according to study publication.

### Treatment-limiting adverse events reported under MTX exposure

Table 3 details reported treatment-limiting events reported in published trials, which are the ones most relevant to inform clinical practice. Again, heterogeneity is high, as evident from the Forest plots of all individual limiting AE’s (S6 File). However, the systematic trend to under-reporting in small studies is much less pronounced in these more serious AEs, although, as expected for rare events, most limiting AEs are reported in the trials with larger sample sizes (Fig 3b). Of note, the weighted incidences shown are effectively an over-estimate since they can only be calculated based on those studies reporting any such AE, thus leaving out what could well be absence of their occurrence in the remainder of studies. (This is reflected in the greatly reduced overall number of apparent patient-safety years.) Thus, even though the sum of the individual AE incidences, as listed in Table 3, would appear to be 14.6%, the reported incidence of treatment limiting AE’s across all 20 studies reported is 6.9% across all studies (Table 3, bottom row). Although it is likely that studies not reporting a treatment limiting AE in fact didn’t observe it, we cannot make that assumption. Thus, for example, two studies reported treatment-limiting leukopenia whereas the remainder of studies did not. In addition, as with general AE reporting, the influence of dosing and route of administration on treatment-limiting AEs is unclear. Nevertheless, the published data detailed in Table 3 support two conclusions. First, the spectrum of treatment—limiting AE observed in clinical trials (nausea, abnormal LFT, leucopenia, diarrhoea, headache) more or less mirrors that observed in clinical practice. Second, more than 90% of patients receiving MTX in clinical studies do *not* exhibit any treatment limiting side effects within six months of treatment.

**Table 3:** Treatment limiting AEs occurring under methotrexate treatment^1^.

AE term^4^	Incidence^2^	Range	Studies reporting	Duration^3^ (months)	Safety years^5^
N/V	3.6%	2.2–6.4	2	8 (4–12)	529
Abnormal LFTs	2.8%	0.6–7.4	7	9 (4–12)	1186
Leucopenia	2.0%	2–2	2	12 (6–18)	102
GI	1.1%	0.8–1.7	2	12	680
Hepatitis	0.8%	0.6–0.9	2	8 (4–12)	200
Pneumonia	0.8%	0.5–1.1	2	8 (4–12)	213
Int. pneumonitis	0.7%	0.5–1	4	12 (12–24)	1047
MI	0.6%	0.5–0.7	2	11.5 (11–12)	449
Alopecia	0.5%	0.2–1.1	2	12	680
Any Tx lim^6^	6.9%	1.6–28	19	6 (3–24)	2738

^1^ Data shown include adverse events reported in at least two independent studies with >200 total patient safety years and with a weighted incidence of > 0.1%. The full data set containing all reviewed studies is provided in the Supplement. See note 1 for Table 2 above for calculation of weighted averages.

^2^ Incidence shown is a weighted incidence to account for the variability of patient numbers across studies, as detailed in Methods.

^3^ Median duration across studies reporting an AE.

^4^ Abbreviations: N/V—nausea and vomiting; GI—any gastrointestinal event; Int. pneumonitis—interstitial pneumonitis, MI—myocardial infarction.

^5^ Total patient safety years underlying each AE. The average number of safety years per study is contained the Supplement (S2 Table).

^6^ Any Tx lim—all treatment limiting adverse events as reported in each study.

### Meta-analysis of efficacy studies on MTX in psoriasis

Table 4 graphically summarises the bias assessment carried out for studies assessed for efficacy assessment. The overall patient numbers as well as the per-study patient numbers are strikingly low compared to studies included for safety variables (937 vs 5995, see above, Table 1). Even this small number of patients mostly consists of patients receiving MTX as comparator treatment for other drugs. For the purpose of efficacy analysis, we did retain partially blinded or un-blinded studies (Table 4, marked by ‘x’). The rationale behind this is that, in contrast to the analysis reported in [1], we did not aim at defining comparative efficacy of MTX versus other treatments but rather at defining an efficacy outcome informative for routine practice under real world conditions. Therefore, any potential bias *in favour* of MTX introduced by including incompletely blinded studies is more than likely offset by the inherent bias *against* MTX by omission of two key variables used in routine care to optimise response to MTX, namely individual dose adjustment as well as alteration of route of administration. Two studies found to have significant issues were omitted from further analysis (Table 4). Taken together, the published database allowing any assessment of MTX efficacy in psoriasis is strikingly small despite decades of clinical use.

**Table 4:** Assessment of bias-profile in published trials on MTX efficacy in psoriasis^1^.

Study^1^													
	A	B	C^3^	D	E	F	G	H	I	J	K	L	M
# of patients^2^	18	215	51	27	37	15	43	19	22	202	15	163	110
Incomplete blinding	x	x		x		x		x	x				
Randomization	1	1	1	1	1	1	1	1	1	3	2	3	1
Group allocation	2	2	1	1	1	2	3	2	2	2	2	1	1
Participant blinding	2	2	1	2	1	2	1	2	2	2	2	1	1
Assessor blinding	2	2	1	2	1	1	1	1	1	3	2	1	1
Data completeness	3	1	3	3	3	1	1	3	3	3	3	1	1
Outcome reporting	1	1	1	1	1	1	1	1	1	2	2	1	1
Other bias	1	1	1	1	1	1	1	1	1	1	1	1	1
DLQI reported^4^		x			x				x			x	

^1^ Clinical trials reporting efficacy data of MTX in psoriasis were identified by literature search as detailed in Methods. Studies are as follows: A Akhyani 2010, B Barker 2011, C Dogra 2012, D Fallah Arani 2011, E Flystrom 2007, F Goldminz¬ 2015, G Heyendael 2003, H Ho 2010, I Lajevardi 2015, J Radmenesh 2011, K Ranjan 2007, L Reich 2011, M Saurat 2007 Bias-profile was analysed using the Cochrane Risk of Bias tool (see Methods). Coding is: 1—low risk, 2—high risk, 3—unclear risk. Studies Radmenesh 2011 and Ranjan 2007 were omitted from further analysis based on unacceptably high bias profile.

^2^ Shown are the number of patients reported in each study allocated to the methotrexate—only arm.

^3^ This study reports two separate sub-cohorts at different dosing levels, respectively.

^4^ DLQI—Dermatology Life Quality Index [51]

### Efficacy outcomes of MTX in psoriasis

Despite the obvious limitations of limited study numbers, divergent study design, non-uniform outcome reporting, and small patient numbers, we performed a meta-analysis of treatment efficacy based on the only variable that was accessible across all studies analysed: the percentage of patients achieving 75% reduction of PASI from baseline (PASI75). Fig 2 graphically summarises the PASI75 reported in the MTX-only treatment arm at 12 or 16 weeks, respectively, in each of the studies analysed. As evident from the figure, there is notable heterogeneity between studies (I^2^ = 92.7%). Dose range as underlying factor is unlikely, given that many studies employed a flexible dose increase scheme, covering a brought overall range (for details, see S4 File). We were also unable to identify any other systematic potential causes, e.g. sample size, dosing scheme. Therefore, in the absence of any discernable factor accounting for heterogeneity, the figure displays a random effects model. The pooled PASI75 estimate calculated across all studies yielded at PASI75 of 45.2% (95% confidence interval 34.1%–60.0%) compared to a calculated PASI75 of 4.4% for placebo (95% confidence interval 3.5%–5.6%) (S4 Table). This yields a relative risk of 10.2 (95% confidence interval 7.1–14.7). Bearing in mind the caveats detailed above, we conclude that, across published MTX studies on psoriasis, approximately 40% of patients achieve PASI75 between weeks 12–16.

## Discussion

### Summary of evidence

Few would argue that methotrexate is as effective as modern biologics for the treatment of moderate to severe psoriasis. Its comparative inferiority has previously been summarised in an in-depth meta-analysis [1]. However, when trying to determine just how effective it is, or just how common side effects occur, we are confronted with a limited database. In terms of safety, the present analysis uncovers a systematic bias toward under-reporting of adverse effects in studies of small sample size (S4 File). This observation adds support to our inclusion of studies using the same dose range as used for psoriasis in similar indications (i.e. chronic inflammatory, non-cancer indications). If one were to restrict safety analysis to studies on psoriasis, many of the adverse effects clinically relevant for psoriasis patients simply would not be detectable due to limiting cohort sizes. Moreover, one inherent overall limitation is the duration of published studies. In general, these are too short to detect delayed-onset adverse effects. Thus, only real-world data would be able to identify frequencies of late-onset adverse effects occurring with long-term dosing.

The overall spectrum of adverse effects mirrors that observed in routine practice. Regarding treatment limiting AE’s it is worth noting that, despite common perception, the most common limiting effects, LFT aberration and nausea, are rare. The latter was only reported in 2 / 20 trials, somewhat reducing the statistical significance of the actual incidence. Limiting LFT aberration also occurs infrequently (3.1% of patients, reported in 8 out of 22 studies). These findings are in agreement with a meta-analysis of liver-injury in methotrexate users, which found that patients on methotrexate are not at increased risk of liver cirrhosis or liver failure [46].

In terms of efficacy, the present meta-analysis finds that approximately 40% of patients achieve a PASI75 by 12 weeks of treatment. As with adverse effect, the main issue evident from the data is the marked heterogeneity, the spread ranging between 9% and 92% of patients reaching this threshold (Fig 2). One factor underlying this heterogeneity would appear to be study design, for example, single-blinded design (see Table 3). Other possible causes are ethnic heterogeneity between patient cohorts, precise dosing scheme, or confounding metabolic factors. Thus, pre-treatment calcium levels were most recently found to be a predictor of treatment response [47]. Clearly, this number is much lower than that achieved by modern biologics, as detailed in [1]. Nonetheless, it means that many psoriasis patients requiring systemic therapy are likely to benefit from methotrexate, even more so, as flexible dose adjustment and adaptation of route of dosing are applied under real-world conditions which would be expected to increase efficacy and reduce limiting adverse events.

Most recently, both FDA and EMA granted a licence for the biologic secukinumab for psoriasis. Significantly, the licence was granted as first-line systemic treatment (www.fda.gov/downloads/drugs/drugsafety/ucm433352.pdf), in principle enabling clinicians to choose this costly drug *instead* of the widely used first-line low cost option of MTX. In light of development such as this one, it will become increasingly important for health care providers to understand if reasonable efforts are being made to employ cost-effective treatments wherever safe and effective. To this end, benchmarks need to be developed under real world conditions in order to allow audit of treatment outcomes. To assist with this, we developed a documentation tool (S7 File). Since staff time is limiting in most routine clinical settings, the tool has been designed to be completed by patients, with clinician input limited to only recording of global psoriasis state (PGA) and LFT aberration. In terms of safety outcomes, the tool exploits the fact that most adverse effect in fact represent *patient reported outcomes* (see Tables 2 and 3). In terms of efficacy, since completion of PASI scores is rarely feasible in routine clinics, we selected the much easier to use commonly employed PGA scale. This is informative, since PASI75 closely approximates PGA 0/1 in clinical trial settings [48] and since PASI and PGA exhibit close correlation across all severity bands [49]. The tool is easily adaptable to electronic documentation systems wherever these are used (e.g., using Google-Forms). Aside from audit, this tool should also allow prospective collection of data with minimal selection and reporting bias.

### Limitations

One important limitation of the present report is that, in terms of safety profile, we present two meta-analyses, where the analysis of safety includes indications other than psoriasis. The rationale for this decision is that (i) the primary safety determinant of methotrexate is the dose range employed, which is identical between the non-psoriasis and psoriasis studies and (ii) that no published data have been reported to indicate that the pharmaco-responsiveness between psoriasis and non-psoriasis patients deviates in the general population for any drug, thereby rendering safety data from non-psoriasis trials relevant for psoriasis patients. However, we cannot exclude the theoretical possibility that, with sufficiently large data to hand, the safety profile between psoriasis and non-psoriasis cohorts would diverge.

### Conclusions

The present meta-analysis vividly illustrates and reinforces the limitations of clinical trials as basis of policy and guideline drafting. Compared to biologics, only minimal evidence is available on treatment efficacy of methotrexate in psoriasis. Since this is unlikely to change, given funding constraints for clinical trials, real-world data, although inherently fuzzy, incomplete, and inevitably less well documented, are the sole source for understanding the performance of drugs such as methotrexate in their intended use, e.g. longterm [50].

## Supporting Information

S1 File PRISMA Checklist. (PDF)

S2 File Detailed-outcomes-SAFETY. (XLSX)

S3 File Detailed-outcomes-EFFICACY. (XLSX)

S4 File Forest plots for non-treatment limiting AE’s with high heterogeneity. (TIF)

S5 File Forest plots for non-treatment limiting AE’s with low heterogeneity. (TIF)

S6 File Forest plots for treatment limiting AE’s. (TIF)

S7 File MTX Treatment Documentation Tool. (DOCX)

S1 Table Adverse effects associated with MTX treatments in published trials. (DOCX)

S2 Table Treatment limiting AE’s occurring under methotrexate treatment. (DOCX)

S3 Table Summary safety outcome statistics from published data. (DOCX)

S4 Table Data on methotrexate placebo arm—allocated patients. (DOCX)

## References

[1] Schmitt J, Rosumeck S, Thomaschewski G, Sporbeck B, Haufe E, Nast A. Efficacy and safety of systemic treatments for moderate-to-severe psoriasis: meta-analysis of randomized controlled trials. The British journal of dermatology. 2014;170(2):274–303. 10.1111/bjd.12663 . 24131260

[2] Baranauskaite A, Raffayova H, Kungurov NV, Kubanova A, Venalis A, Helmle L, et al Infliximab plus methotrexate is superior to methotrexate alone in the treatment of psoriatic arthritis in methotrexate-naive patients: the RESPOND study. Annals of the rheumatic diseases. 2012;71(4):541–8. 10.1136/ard.2011.152223 21994233 PMC3298666

[3] Barker J, Hoffmann M, Wozel G, Ortonne JP, Zheng H, van Hoogstraten H, et al Efficacy and safety of infliximab vs. methotrexate in patients with moderate-to-severe plaque psoriasis: results of an open-label, active-controlled, randomized trial (RESTORE1). The British journal of dermatology. 2011;165(5):1109–17. . 21910713 10.1111/j.1365-2133.2011.10615.x

[4] Bathon JM, Martin RW, Fleischmann RM, Tesser JR, Schiff MH, Keystone EC, et al A comparison of etanercept and methotrexate in patients with early rheumatoid arthritis. The New England journal of medicine. 2000;343(22):1586–93. 10.1056/NEJM200011303432201 . 11096165

[5] Braun J, Kastner P, Flaxenberg P, Wahrisch J, Hanke P, Demary W, et al Comparison of the clinical efficacy and safety of subcutaneous versus oral administration of methotrexate in patients with active rheumatoid arthritis: results of a six-month, multicenter, randomized, double-blind, controlled, phase IV trial. Arthritis and rheumatism. 2008;58(1):73–81. 10.1002/art.23144 . 18163521

[6] Breedveld FC, Weisman MH, Kavanaugh AF, Cohen SB, Pavelka K, van Vollenhoven R, et al The PREMIER study: A multicenter, randomized, double-blind clinical trial of combination therapy with adalimumab plus methotrexate versus methotrexate alone or adalimumab alone in patients with early, aggressive rheumatoid arthritis who had not had previous methotrexate treatment. Arthritis and rheumatism. 2006;54(1):26–37. 10.1002/art.21519 . 16385520

[7] Capell HA, Madhok R, Porter DR, Munro RA, McInnes IB, Hunter JA, et al Combination therapy with sulfasalazine and methotrexate is more effective than either drug alone in patients with rheumatoid arthritis with a suboptimal response to sulfasalazine: results from the double-blind placebo-controlled MASCOT study. Annals of the rheumatic diseases. 2007;66(2):235–41. 10.1136/ard.2006.057133 16926184 PMC1798490

[8] Cohen S, Cannon GW, Schiff M, Weaver A, Fox R, Olsen N, et al Two-year, blinded, randomized, controlled trial of treatment of active rheumatoid arthritis with leflunomide compared with methotrexate. Utilization of Leflunomide in the Treatment of Rheumatoid Arthritis Trial Investigator Group. Arthritis and rheumatism. 2001;44(9):1984–92. 10.1002/1529-0131(200109)44:9<1984::AID-ART346>3.0.CO;2-B . 11592358

[9] Cohen SB, Cheng TT, Chindalore V, Damjanov N, Burgos-Vargas R, Delora P, et al Evaluation of the efficacy and safety of pamapimod, a p38 MAP kinase inhibitor, in a double-blind, methotrexate-controlled study of patients with active rheumatoid arthritis. Arthritis and rheumatism. 2009;60(2):335–44. 10.1002/art.24266 . 19180516

[10] De Groot K, Rasmussen N, Bacon PA, Tervaert JW, Feighery C, Gregorini G, et al Randomized trial of cyclophosphamide versus methotrexate for induction of remission in early systemic antineutrophil cytoplasmic antibody-associated vasculitis. Arthritis and rheumatism. 2005;52(8):2461–9. 10.1002/art.21142 . 16052573

[11] Dougados M, Combe B, Cantagrel A, Goupille P, Olive P, Schattenkirchner M, et al Combination therapy in early rheumatoid arthritis: a randomised, controlled, double blind 52 week clinical trial of sulphasalazine and methotrexate compared with the single components. Annals of the rheumatic diseases. 1999;58(4):220–5. 10364900 10.1136/ard.58.4.220 PMC1752864

[12] Emery P, Breedveld FC, Hall S, Durez P, Chang DJ, Robertson D, et al Comparison of methotrexate monotherapy with a combination of methotrexate and etanercept in active, early, moderate to severe rheumatoid arthritis (COMET): a randomised, double-blind, parallel treatment trial. Lancet. 2008;372(9636):375–82. 10.1016/S0140-6736(08)61000-4 . 18635256

[13] Emery P, Breedveld FC, Lemmel EM, Kaltwasser JP, Dawes PT, Gomor B, et al A comparison of the efficacy and safety of leflunomide and methotrexate for the treatment of rheumatoid arthritis. Rheumatology. 2000;39(6):655–65. . 10888712 10.1093/rheumatology/39.6.655

[14] Feagan BG, Rochon J, Fedorak RN, Irvine EJ, Wild G, Sutherland L, et al Methotrexate for the treatment of Crohn's disease. The North American Crohn's Study Group Investigators. The New England journal of medicine. 1995;332(5):292–7. 10.1056/NEJM199502023320503 . 7816064

[15] Janagond AB, Kanwar AJ, Handa S. Efficacy and safety of systemic methotrexate vs. acitretin in psoriasis patients with significant palmoplantar involvement: a prospective, randomized study. Journal of the European Academy of Dermatology and Venereology: JEADV. 2013;27(3):e384–9. 10.1111/jdv.12004 . 23066720

[16] Jones G, Sebba A, Gu J, Lowenstein MB, Calvo A, Gomez-Reino JJ, et al Comparison of tocilizumab monotherapy versus methotrexate monotherapy in patients with moderate to severe rheumatoid arthritis: the AMBITION study. Annals of the rheumatic diseases. 2010;69(1):88–96. 10.1136/ard.2008.105197 19297346 PMC3747519

[17] Kavanaugh A, Fleischmann RM, Emery P, Kupper H, Redden L, Guerette B, et al Clinical, functional and radiographic consequences of achieving stable low disease activity and remission with adalimumab plus methotrexate or methotrexate alone in early rheumatoid arthritis: 26-week results from the randomised, controlled OPTIMA study. Annals of the rheumatic diseases. 2013;72(1):64–71. 10.1136/annrheumdis-2011-201247 22562973 PMC3551224

[18] Kingsley GH, Kowalczyk A, Taylor H, Ibrahim F, Packham JC, McHugh NJ, et al A randomized placebo-controlled trial of methotrexate in psoriatic arthritis. Rheumatology. 2012;51(8):1368–77. 10.1093/rheumatology/kes001 22344575 PMC3397466

[19] Klareskog L, van der Heijde D, de Jager JP, Gough A, Kalden J, Malaise M, et al Therapeutic effect of the combination of etanercept and methotrexate compared with each treatment alone in patients with rheumatoid arthritis: double-blind randomised controlled trial. Lancet. 2004;363(9410):675–81. 10.1016/S0140-6736(04)15640-7 . 15001324

[20] Lee EB, Fleischmann R, Hall S, Wilkinson B, Bradley JD, Gruben D, et al Tofacitinib versus methotrexate in rheumatoid arthritis. The New England journal of medicine. 2014;370(25):2377–86. 10.1056/NEJMoa1310476 . 24941177

[21] Lerndal T, Svensson B. A clinical study of CPH 82 vs methotrexate in early rheumatoid arthritis. Rheumatology. 2000;39(3):316–20. . 10788542 10.1093/rheumatology/39.3.316

[22] Lu LJ, Bao CD, Dai M, Teng JL, Fan W, Du F, et al Multicenter, randomized, double-blind, controlled trial of treatment of active rheumatoid arthritis with T-614 compared with methotrexate. Arthritis and rheumatism. 2009;61(7):979–87. 10.1002/art.24643 . 19565542

[23] Reich K, Langley RG, Papp KA, Ortonne JP, Unnebrink K, Kaul M, et al A 52-week trial comparing briakinumab with methotrexate in patients with psoriasis. The New England journal of medicine. 2011;365(17):1586–96. 10.1056/NEJMoa1010858 . 22029980

[24] Rigby W, Tony HP, Oelke K, Combe B, Laster A, von Muhlen CA, et al Safety and efficacy of ocrelizumab in patients with rheumatoid arthritis and an inadequate response to methotrexate: results of a forty-eight-week randomized, double-blind, placebo-controlled, parallel-group phase III trial. Arthritis and rheumatism. 2012;64(2):350–9. 10.1002/art.33317 . 21905001

[25] Saurat JH, Stingl G, Dubertret L, Papp K, Langley RG, Ortonne JP, et al Efficacy and safety results from the randomized controlled comparative study of adalimumab vs. methotrexate vs. placebo in patients with psoriasis (CHAMPION). The British journal of dermatology. 2008;158(3):558–66. 10.1111/j.1365-2133.2007.08315.x . 18047523

[26] Smolen J, Landewe RB, Mease P, Brzezicki J, Mason D, Luijtens K, et al Efficacy and safety of certolizumab pegol plus methotrexate in active rheumatoid arthritis: the RAPID 2 study. A randomised controlled trial. Annals of the rheumatic diseases. 2009;68(6):797–804. 10.1136/ard.2008.101659 19015207 PMC2674556

[27] Smolen JS, Emery P, Fleischmann R, van Vollenhoven RF, Pavelka K, Durez P, et al Adjustment of therapy in rheumatoid arthritis on the basis of achievement of stable low disease activity with adalimumab plus methotrexate or methotrexate alone: the randomised controlled OPTIMA trial. Lancet. 2014;383(9914):321–32. 10.1016/S0140-6736(13)61751-1 . 24168956

[28] St Clair EW, van der Heijde DM, Smolen JS, Maini RN, Bathon JM, Emery P, et al Combination of infliximab and methotrexate therapy for early rheumatoid arthritis: a randomized, controlled trial. Arthritis and rheumatism. 2004;50(11):3432–43. 10.1002/art.20568 . 15529377

[29] Strand V, Cohen S, Schiff M, Weaver A, Fleischmann R, Cannon G, et al Treatment of active rheumatoid arthritis with leflunomide compared with placebo and methotrexate. Leflunomide Rheumatoid Arthritis Investigators Group. Archives of internal medicine. 1999;159(21):2542–50. . 10573044 10.1001/archinte.159.21.2542

[30] Takeuchi T, Miyasaka N, Zang C, Alvarez D, Fletcher T, Wajdula J, et al A phase 3 randomized, double-blind, multicenter comparative study evaluating the effect of etanercept versus methotrexate on radiographic outcomes, disease activity, and safety in Japanese subjects with active rheumatoid arthritis. Modern rheumatology / the Japan Rheumatism Association. 2013;23(4):623–33. 10.1007/s10165-012-0742-6 . 23011358

[31] Tanaka Y, Harigai M, Takeuchi T, Yamanaka H, Ishiguro N, Yamamoto K, et al Golimumab in combination with methotrexate in Japanese patients with active rheumatoid arthritis: results of the GO-FORTH study. Annals of the rheumatic diseases. 2012;71(6):817–24. 10.1136/ard.2011.200317 22121129 PMC3372319

[32] van Dongen H, van Aken J, Lard LR, Visser K, Ronday HK, Hulsmans HM, et al Efficacy of methotrexate treatment in patients with probable rheumatoid arthritis: a double-blind, randomized, placebo-controlled trial. Arthritis and rheumatism. 2007;56(5):1424–32. 10.1002/art.22525 . 17469099

[33] Westhovens R, Robles M, Ximenes AC, Nayiager S, Wollenhaupt J, Durez P, et al Clinical efficacy and safety of abatacept in methotrexate-naive patients with early rheumatoid arthritis and poor prognostic factors. Annals of the rheumatic diseases. 2009;68(12):1870–7. 10.1136/ard.2008.101121 19124524 PMC2770104

[34] Yamamoto K, Takeuchi T, Yamanaka H, Ishiguro N, Tanaka Y, Eguchi K, et al Efficacy and safety of certolizumab pegol plus methotrexate in Japanese rheumatoid arthritis patients with an inadequate response to methotrexate: the J-RAPID randomized, placebo-controlled trial. Modern rheumatology / the Japan Rheumatism Association. 2014;24(5):715–24. 10.3109/14397595.2013.864224 . 24313916

[35] Dhir V, Singla M, Gupta N, Goyal P, Sagar V, Sharma A, et al Randomized controlled trial comparing 2 different starting doses of methotrexate in rheumatoid arthritis. Clinical therapeutics. 2014;36(7):1005–15. 10.1016/j.clinthera.2014.05.063 . 24976447

[36] Akhyani M, Chams-Davatchi C, Hemami MR, Fateh S. Efficacy and safety of mycophenolate mofetil vs. methotrexate for the treatment of chronic plaque psoriasis. Journal of the European Academy of Dermatology and Venereology: JEADV. 2010;24(12):1447–51. 10.1111/j.1468-3083.2010.03667.x . 20384673

[37] Dogra S, Krishna V, Kanwar AJ. Efficacy and safety of systemic methotrexate in two fixed doses of 10 mg or 25 mg orally once weekly in adult patients with severe plaque-type psoriasis: a prospective, randomized, double-blind, dose-ranging study. Clinical and experimental dermatology. 2012;37(7):729–34. 10.1111/j.1365-2230.2012.04440.x . 22830389

[38] Fallah Arani S, Neumann H, Hop WC, Thio HB. Fumarates vs. methotrexate in moderate to severe chronic plaque psoriasis: a multicentre prospective randomized controlled clinical trial. The British journal of dermatology. 2011;164(4):855–61. 10.1111/j.1365-2133.2010.10195.x . 21175564

[39] Flytstrom I, Stenberg B, Svensson A, Bergbrant IM. Methotrexate vs. ciclosporin in psoriasis: effectiveness, quality of life and safety. A randomized controlled trial. The British journal of dermatology. 2008;158(1):116–21. 10.1111/j.1365-2133.2007.08284.x . 17986302

[40] Heydendael VM, Spuls PI, Opmeer BC, de Borgie CA, Reitsma JB, Goldschmidt WF, et al Methotrexate versus cyclosporine in moderate-to-severe chronic plaque psoriasis. The New England journal of medicine. 2003;349(7):658–65. 10.1056/NEJMoa021359 . 12917302

[41] Lajevardi V, Hallaji Z, Daklan S, Abedini R, Goodarzi A, Abdolreza M. The efficacy of methotrexate plus pioglitazone vs. methotrexate alone in the management of patients with plaque-type psoriasis: a single-blinded randomized controlled trial. International journal of dermatology. 2015;54(1):95–101. 10.1111/ijd.12585 . 25209868

[42] Ho SG, Yeung CK, Chan HH. Methotrexate versus traditional Chinese medicine in psoriasis: a randomized, placebo-controlled trial to determine efficacy, safety and quality of life. Clinical and experimental dermatology. 2010;35(7):717–22. 10.1111/j.1365-2230.2009.03693.x . 19925489

[43] Radmanesh M, Rafiei B, Moosavi ZB, Sina N. Weekly vs. daily administration of oral methotrexate (MTX) for generalized plaque psoriasis: a randomized controlled clinical trial. International journal of dermatology. 2011;50(10):1291–3. 10.1111/j.1365-4632.2011.04967.x . 21950300

[44] Ranjan N, Sharma NL, Shanker V, Mahajan VK, Tegta GR. Methotrexate versus hydroxycarbamide (hydroxyurea) as a weekly dose to treat moderate-to-severe chronic plaque psoriasis: a comparative study. The Journal of dermatological treatment. 2007;18(5):295–300. 10.1080/09546630701499291 . 17852635

[45] Higgins JP, Altman DG, Gotzsche PC, Juni P, Moher D, Oxman AD, et al The Cochrane Collaboration's tool for assessing risk of bias in randomised trials. Bmj. 2011;343:d5928 10.1136/bmj.d5928 22008217 PMC3196245

[46] Conway R, Low C, Coughlan RJ, O'Donnell MJ, Carey JJ. Risk of liver injury among methotrexate users: A meta-analysis of randomised controlled trials. Seminars in arthritis and rheumatism. 2015;45(2):156–62. 10.1016/j.semarthrit.2015.05.003 . 26088004

[47] Zhai Z, Chen L, Yang H, Yan J, Wang C, Yang J, et al Can pretreatment serum calcium level predict the efficacy of methotrexate in the treatment of severe plaque psoriasis? Journal of the American Academy of Dermatology. 2015;73(6):991–7.e3. 10.1016/j.jaad.2015.08.036 . 26416303

[48] Robinson A, Kardos M, Kimball AB. Physician Global Assessment (PGA) and Psoriasis Area and Severity Index (PASI): why do both? A systematic analysis of randomized controlled trials of biologic agents for moderate to severe plaque psoriasis. Journal of the American Academy of Dermatology. 2012;66(3):369–75. 10.1016/j.jaad.2011.01.022 . 22041254

[49] Cappelleri JC, Bushmakin AG, Harness J, Mamolo C. Psychometric validation of the physician global assessment scale for assessing severity of psoriasis disease activity. Quality of life research: an international journal of quality of life aspects of treatment, care and rehabilitation. 2013;22(9):2489–99. 10.1007/s11136-013-0384-y . 23475691

[50] Moher D, Liberati A, Tetzlaff J, Altman DG, Group P. Preferred reporting items for systematic reviews and meta-analyses: the PRISMA statement. Journal of clinical epidemiology. 2009;62(10):1006–12. 10.1016/j.jclinepi.2009.06.005 . 19631508

[51] Finlay AY, Khan GK. Dermatology Life Quality Index (DLQI)—a simple practical measure for routine clinical use. Clinical and experimental dermatology. 1994;19(3):210–6. . 8033378 10.1111/j.1365-2230.1994.tb01167.x

