# Correction: Safety and Efficacy of Methotrexate in Psoriasis: A Meta-Analysis of Published Trials

**DOI:** 10.1371/journal.pone.0158928

**Published:** 2016-07-05

**Authors:** The PLOS ONE Staff

Fig 1 is incorrect. Please see the correct Fig 1 here. The publisher apologizes for the error.

**Fig 1:**
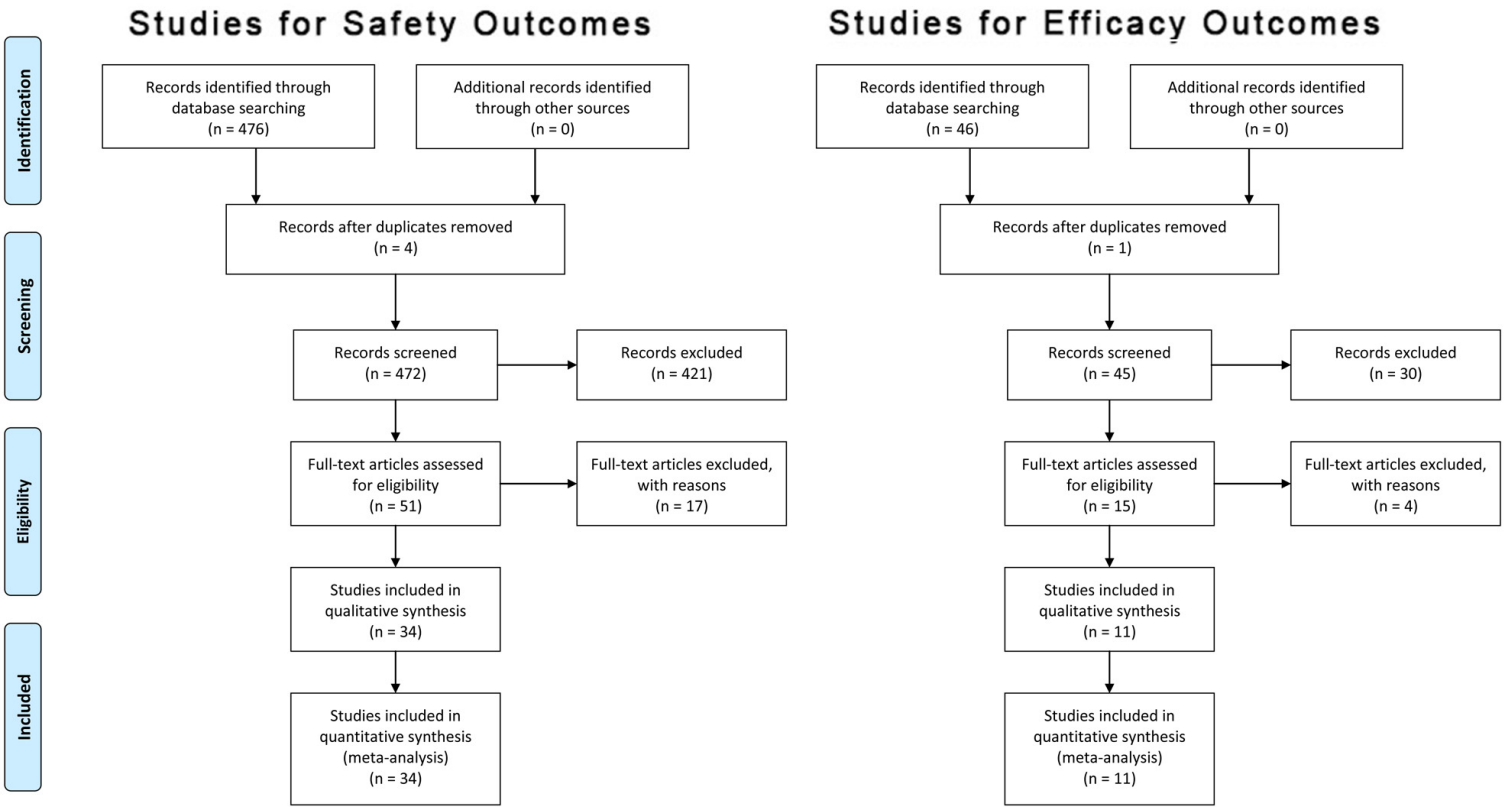
PRISMA flow diagram according to [50] summarising study selection for clinical trials reporting safety (left) and efficacy (right) outcomes, respectively. Search terms employed were: methotrexate [Title] AND (psoriasis [Title] OR arthritis [Title] OR Crohn’s [Title] OR ulcerative colitis [Title] OR ankylosing spondylitis [Title]) AND (trial [Title] OR Study [Title]) for safety studies and: Search: methotrexate [Title] AND psoriasis [Title] AND (trial [Title] OR Study [Title]) for efficacy studies.
