# Supplementary figures and images for: Safety and Efficacy of Methotrexate in Psoriasis: A Meta-Analysis of Published Trials
Source: PLoS One. 2016 May 11;11(5):e0153740. doi: 10.1371/journal.pone.0153740 (PMC4864230; doi:10.1371/journal.pone.0153740)

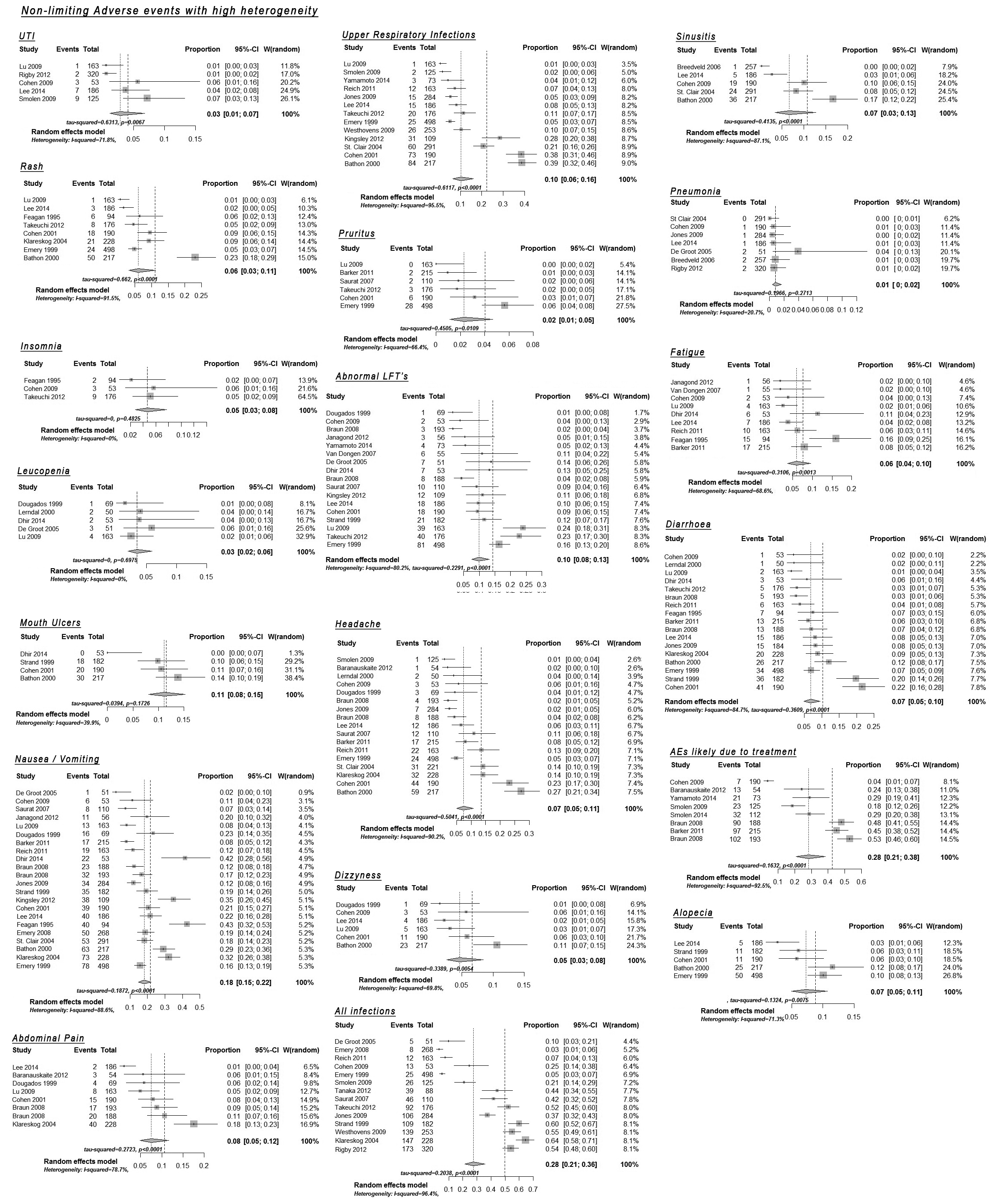

Supplement: S4 File — (TIF) [file pone.0153740.s004.tif]

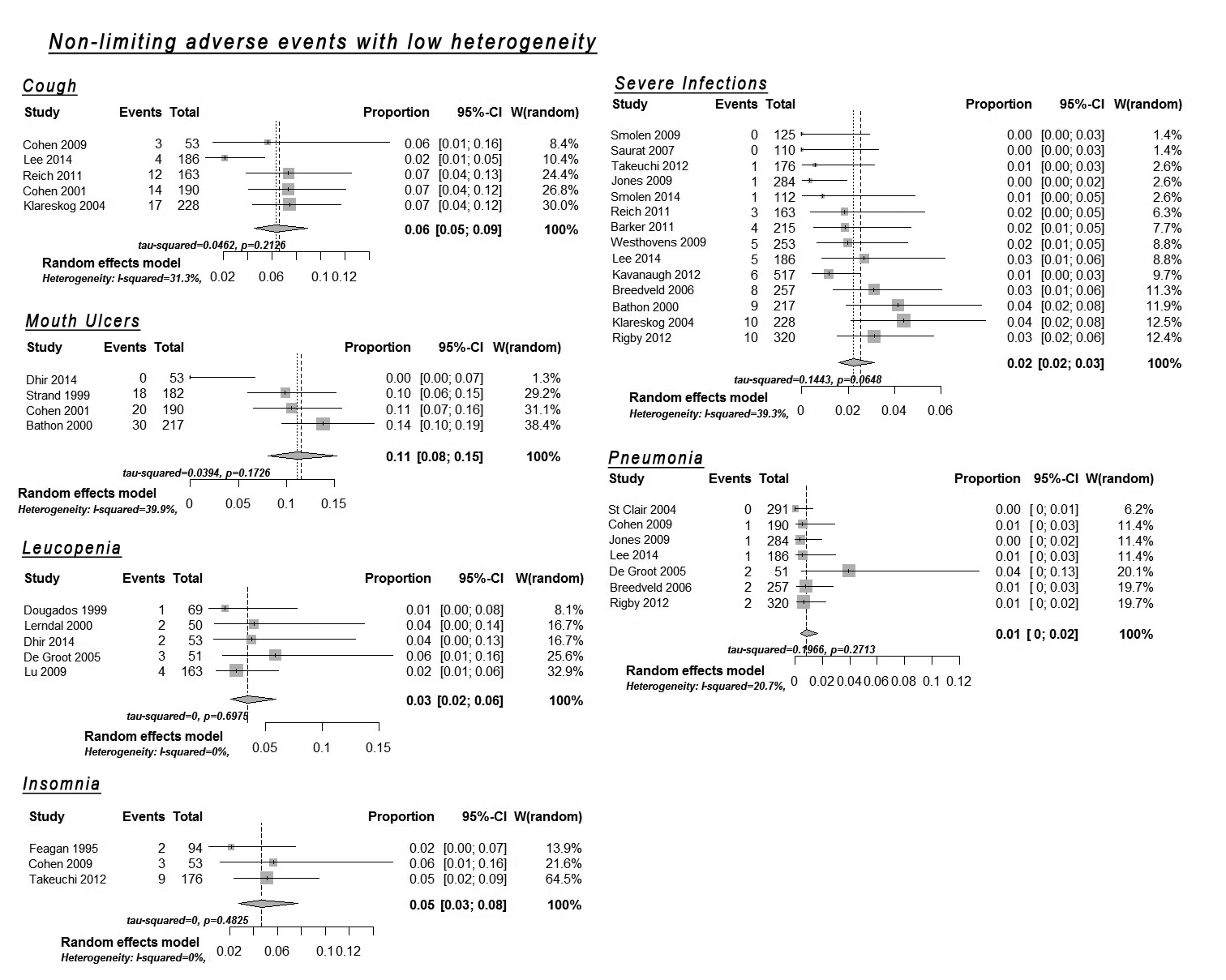

Supplement: S5 File — (TIF) [file pone.0153740.s005.tif]

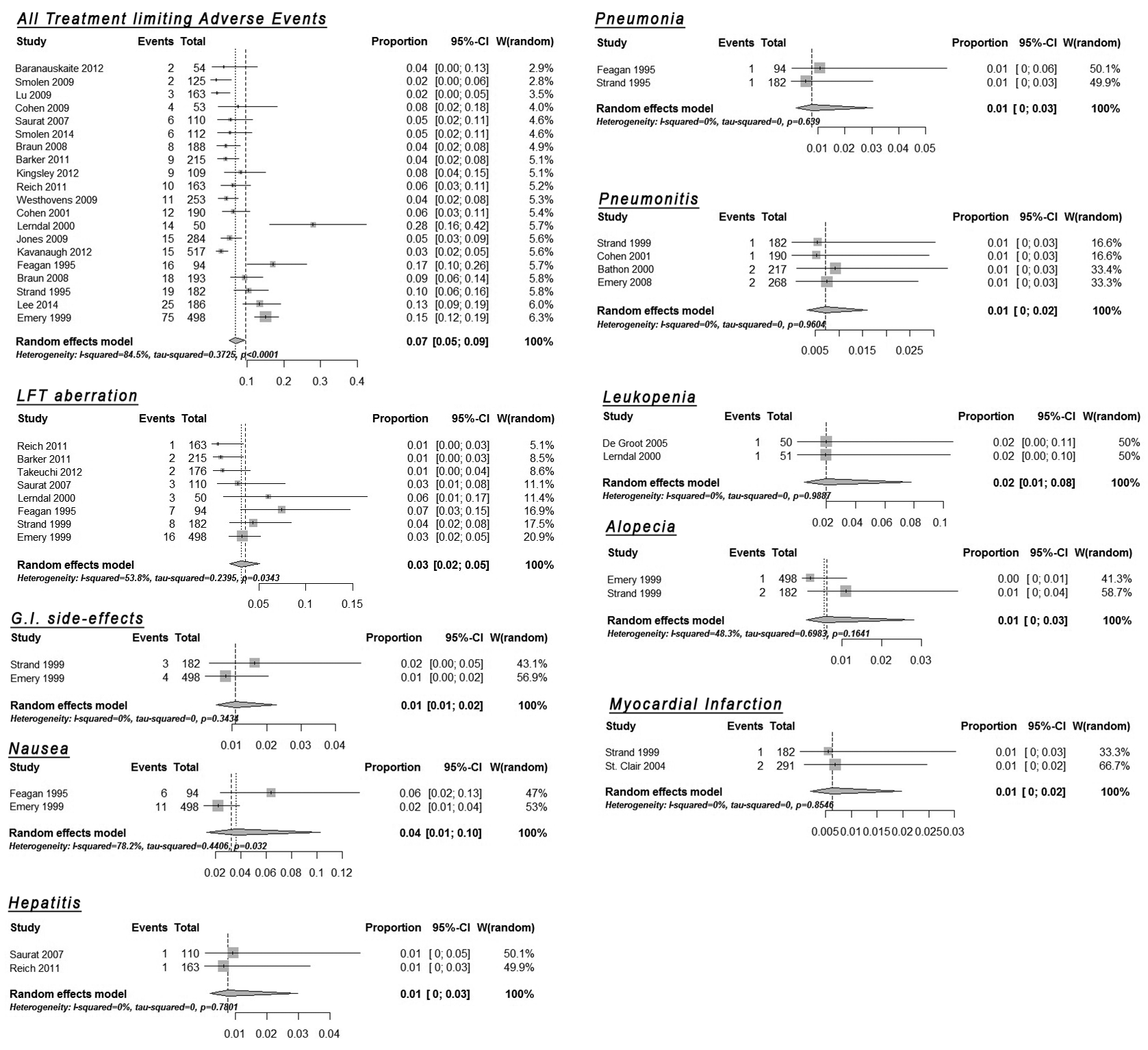

Supplement: S6 File — (TIF) [file pone.0153740.s006.tif]
