# Supplementary material for: Safety and Efficacy of Methotrexate in Psoriasis: A Meta-Analysis of Published Trials
Source: PLoS One. 2016 May 11;11(5):e0153740. doi: 10.1371/journal.pone.0153740 (PMC4864230; doi:10.1371/journal.pone.0153740)
Supplement: S7 File — (DOCX) [file pone.0153740.s007.docx]

**METHOTREXATE TREATMENT DOCUMENTATION**

PATIENT ID

**Before Start:**

Date:

Reason for treatment: Psoriasis ☐ Psoriasis-Arthritis ☐ Other: _______________

Starting dose (after test dose): _______ mg once weekly

I would describe my psoriasis as: Gone / minimal/ mild/ moderate/ bad

I also have / don’t have joint complaints: None at all / mild/ moderate/ bad / very bad

**CLINICIAN TO RECORD**: global assessment clear / minimal / mild / moderate/ severe DLQI recorded ☐

**3 - Month Review:**

Still on Methotrexate Yes ☐ Current dose: _______mg **Changed to injections:** Yes / No

No ☐ I came off, because:

It didn’t work ☐ Because of side effects ☐ Because of other: ________________

I have started to take another regular medicine: Yes / No

Did any of these side effects occur:

|  | Nausea | Sinus | Sore throat/ flu/ bronchitis | Belly ache | Headache | Mouth ulcers | Skin rash | Diarrhoea | Hair loss | Fatigue | Cough | Other |
| --- | --- | --- | --- | --- | --- | --- | --- | --- | --- | --- | --- | --- |
| mild |  |  |  |  |  |  |  |  |  |  |  |  |
| moderate |  |  |  |  |  |  |  |  |  |  |  |  |
| Made me stop |  |  |  |  |  |  |  |  |  |  |  |  |

“**Other**” was: _________________________

My psoriasis now overall is: My joint aches (if present) are now:

Gone / minimal/ mild/ moderate/ bad Gone / mild/ moderate/ bad / very bad

**CLINICIAN TO RECORD**: global assessment: clear / minimal / mild / moderate/ severe DLQI recorded ☐

LFT treatment limiting: ☐

**6 - Month Review:**

PATIENT ID

Still on Methotrexate Yes ☐ Current dose: _______mg **Changed to injections:** Yes / No

No ☐ I came off, because:

It didn’t work ☐ Because of side effects ☐ Because of other: ________________

I have started to take another regular medicine: Yes / No

Did any of these side effects occur:

|  | Nausea | Sinus | Sore throat/ flu/ bronchitis | Belly ache | Headache | Mouth ulcers | Skin rash | Diarrhoea | Hair loss | Fatigue | Cough | Other |
| --- | --- | --- | --- | --- | --- | --- | --- | --- | --- | --- | --- | --- |
| mild |  |  |  |  |  |  |  |  |  |  |  |  |
| moderate |  |  |  |  |  |  |  |  |  |  |  |  |
| Made me stop |  |  |  |  |  |  |  |  |  |  |  |  |

“**Other**” was: _________________________

My psoriasis now overall is: My joint aches (if present) are now:

Gone / minimal/ mild/ moderate/ bad Gone / mild/ moderate/ bad / very bad

**CLINICIAN TO RECORD**: global assessment: clear / minimal / mild / moderate/ severe DLQI recorded ☐ LFT treatment limiting: ☐

**1-year Review:**

Still on Methotrexate Yes ☐ Current dose: _______mg **Changed to injections:** Yes / No

No ☐ I came off, because:

It didn’t work ☐ Because of side effects ☐ Because of other: ________________

I have started to take another regular medicine: Yes / No

Did any of these side effects occur:

|  | Nausea | Sinus | Sore throat/ flu/ bronchitis | Belly ache | Headache | Mouth ulcers | Skin rash | Diarrhoea | Hair loss | Fatigue | Cough | Other |
| --- | --- | --- | --- | --- | --- | --- | --- | --- | --- | --- | --- | --- |
| mild |  |  |  |  |  |  |  |  |  |  |  |  |
| moderate |  |  |  |  |  |  |  |  |  |  |  |  |
| Made me stop |  |  |  |  |  |  |  |  |  |  |  |  |

“**Other**” was: _________________________

My psoriasis now overall is: My joint aches (if present) are now:

Gone / minimal/ mild/ moderate/ bad Gone / mild/ moderate/ bad / very bad

**CLINICIAN TO RECORD**: global assessment: clear / minimal / mild / moderate/ severe DLQI recorded ☐ LFT treatment limiting: ☐
