# Supplementary material for: Safety and Efficacy of Methotrexate in Psoriasis: A Meta-Analysis of Published Trials
Source: PLoS One. 2016 May 11;11(5):e0153740. doi: 10.1371/journal.pone.0153740 (PMC4864230; doi:10.1371/journal.pone.0153740)
Supplement: S1 Table — (DOCX) [file pone.0153740.s008.docx]

**S1 Table. All Adverse effects associated with MTX treatments in published trials**^1^

| AE term^4^ | Incidence^2^ | Range | # of studies | Duration^3^ (months) | Safety years (total) | Safety years (average) | |
| --- | --- | --- | --- | --- | --- | --- | --- |
| All infection | 27.6% | 3 - 65 | 14 | 12 | 2430 | | 174 |
| N/V | 18.2% | 2 - 43 | 22 | 6 | 3117 | | 142 |
| Mouth ulcers | 11.1% | 0 - 14 | 4 | 12 | 792 | | 198 |
| URI | 10.2% | 0.6 - 39 | 13 | 11 | 2424 | | 186 |
| Abnormal LFTs | 10.0% | 1 - 24 | 17 | 6 | 1997 | | 117 |
| Abdominal pain | 7.5% | 1.1 - 18 | 8 | 6 | 1060 | | 133 |
| Headache | 7.3% | 0.8 - 27 | 17 | 6 | 2501 | | 147 |
| Alopecia | 7.3% | 2.7 - 12 | 5 | 12 | 1370 | | 274 |
| Diarrhoea | 6.8% | 1.2 - 22 | 17 | 6 | 2532 | | 149 |
| Sinusitis | 6.6% | 0.2 - 17 | 5 | 12 | 1471 | | 294 |
| Rash | 6.0% | 0.6 - 23 | 8 | 12 | 1705 | | 213 |
| Cough | 6.4% | 2.2 - 7.5 | 5 | 12 | 877 | | 175 |
| Fatigue | 6.1% | 1.8 - 16 | 9 | 6 | 895 | | 99 |
| Dizziness | 4.7% | 1 - 11 | 6 | 9 | 854 | | 142 |
| Insomnia | 4.6% | 2.1 - 5.7 | 3 | 4 | 221 | | 74 |
| Anaemia | 3.4% | 1.8 - 3.8 | 2 | 4.5 | 107 | | 54 |
| Leucopenia | 3.4% | 1 - 5.9 | 5 | 6 | 265 | | 53 |
| UTI | 2.9% | 0.6 - 7.2 | 5 | 6 | 537 | | 107 |
| Pruritus | 2.3% | 0 - 5.6 | 6 | 9 | 1271 | | 212 |
| Allergic reaction | 2.0% | 2 | 1 | 15 | 77 | | 77 |
| AI event | 2.0% | 2 | 1 | 12 | 253 | | 253 |
| Severe infection | 1.4% | 0 - 4.4 | 14 | 11.5 | 2711 | | 194 |
| Bacteraemia | 1.3% | 0.2 - 3.2 | 2 | 15 | 577 | | 289 |
| All malignancy | 1.2% | 0 - 2 | 11 | 12 | 2465 | | 224 |
| RFA | 0.8% | 0.7 - 1.9 | 2 | 8 | 286 | | 143 |
| Pneumonia | 0.8% | 0 - 3.9 | 7 | 11 | 1760 | | 251 |
| Breast cancer | 0.05% | 0 - 1.1 | 11 | 12 | 2465 | | 224 |
| Prostate cancer | 0.04% | 0 - 0.6 | 11 | 12 | 2465 | | 224 |
| Lymphoma | 0.03% | 0 - 0.6 | 11 | 12 | 2465 | | 224 |
| Melanoma | 0.02% | 0 - 0.4 | 11 | 12 | 2465 | | 224 |
| Colon cancer | 0.02% | 0 - 0.3 | 11 | 12 | 2465 | | 224 |
| BCC | 0.02% | 0 - 0.3 | 11 | 12 | 2465 | | 224 |
| SCC | 0.02% | 0 - 0.3 | 11 | 12 | 2465 | | 224 |
| Cholangiocarcinoma | 0.01% | 0 - 0.9 | 11 | 12 | 2465 | | 224 |
| AELTX^5^ | 28.3% | 3.7 - 53 | 8 | 6 | 968 | | 121 |

^1^Data shown includes all adverse events recorded across all studies.

^2^Incidence shown is a weighted incidence to account for the variability of patient numbers across studies, as detailed in Methods.

^3^Median duration across studies reporting an AE.

^4^Abbreviations: N/V - nausea and vomiting; URI - upper respiratory infection; abnormal LFT - abnormal liver function test result; UTI - urinary tract infection; AI event - autoimmune event; RFA - renal function alteration; BCC - basal cell carcinoma; SCC - squamous cell carcinoma

^5^AELTX - all adverse events judged as likely due to treatment according to study publication.
