# Supplementary material for: Safety and Efficacy of Methotrexate in Psoriasis: A Meta-Analysis of Published Trials
Source: PLoS One. 2016 May 11;11(5):e0153740. doi: 10.1371/journal.pone.0153740 (PMC4864230; doi:10.1371/journal.pone.0153740)
Supplement: S2 Table — (DOCX) [file pone.0153740.s009.docx]

**S2 Table. All treatment limiting AE’s occurring under methotrexate treatment**^1^

| AE term^4^ | Incidence^2^ | Range | # of studies | Duration^3^ (months) | | Safety years (total) | Safety years (average) | |
| --- | --- | --- | --- | --- | --- | --- | --- | --- |
| N/V | 3.6% | 2.2 - 6.4 | 2 | 8 | 529 | | | 265 |
| Abnormal LFT | 2.8% | 0.6 - 7.4 | 7 | 9 | 1186 | | | 169 |
| Allergic reaction | 2.0% | 2 | 1 | 18 | 77 | | | 77 |
| Leucopenia | 2.0% | 2 - 2 | 2 | 12 | 102 | | | 51 |
| GI | 1.1% | 0.8 - 1.7 | 2 | 12 | 680 | | | 340 |
| Abdominal pain | 1.0% | 0.9 - 1.1 | 2 | 4 | 68 | | | 34 |
| Headache | 1.0% | 1 | 1 | 12 | 498 | | | 498 |
| Optic neuritis | 1.0% | 0.9 - 1.1 | 2 | 4 | 68 | | | 34 |
| Pneumonia | 0.8% | 0.5 - 1.1 | 2 | 8 | 213 | | | 107 |
| Diarrhoea | 0.8% | 0.8 | 1 | 12 | 498 | | | 498 |
| Hepatitis | 0.8% | 0.6 - 0.9 | 2 | 8 | 200 | | | 100 |
| Int. Pneumonitis | 0.7% | 0.5 - 1 | 4 | 12 | 1047 | | | 262 |
| Rash | 0.7% | 0 - 2 | 4 | 9 | 736 | | | 184 |
| MI | 0.6% | 0.5 - 0.7 | 2 | 11.5 | 449 | | | 225 |
| Alopecia | 0.5% | 0.2 - 1.1 | 2 | 12 | 680 | | | 340 |
| Mouth ulcers | 0.2% | 0.2 | 1 | 12 | 498 | | | 498 |
| Any Tx lim^5^ | 6.9% | 1.6 - 28 | 19 | 6 | 2738 | | | 144 |

^1^Data shown include all adverse events across all studies

^2^Incidence shown is a weighted incidence to account for the variability of patient numbers across studies, as detailed in Methods.

^3^Median duration across studies reporting an AE.

^4^Abbreviations: N/V - nausea and vomiting; abnormal LFT - abnormal liver function test result; GI - any gastrointestinal event; Int. pneumonitis - interstitial pneumonitis; MI - myocardial infarction.

^5^Any Tx lim - all treatment limiting adverse events as documented across studies.
