# Supplementary material for: Safety and Efficacy of Methotrexate in Psoriasis: A Meta-Analysis of Published Trials
Source: PLoS One. 2016 May 11;11(5):e0153740. doi: 10.1371/journal.pone.0153740 (PMC4864230; doi:10.1371/journal.pone.0153740)
Supplement: S3 Table — (DOCX) [file pone.0153740.s010.docx]

**S3 Table. Summary safety outcome statistics from published data**^1^

| AE term^3^ | Incidence^1^ | Range | # of studies | | | Duration^2^ (months) | Safety years (total) | Safety years (average) |
| --- | --- | --- | --- | --- | --- | --- | --- | --- |
| Any AE | 67.1% | 25 - 91.8 | | 25 | 6 | | 2901 | 116 |
| SAE | 6.3% | 0.6 - 18.9 | | 23 | 6 | | 3595 | 156 |
| Death | 0.2% | 0 - 3.9 | | 25 | 6 | | 4187 | 167 |

^1^Incidence shown is a weighted incidence to account for the variability of patient numbers across studies, as detailed in Methods.

^2^Median duration across studies reporting an AE.

^3^Abbreviations: Any AE - any adverse effect; SAE - severe adverse effect.
