# Supplementary material for: Safety and Efficacy of Methotrexate in Psoriasis: A Meta-Analysis of Published Trials
Source: PLoS One. 2016 May 11;11(5):e0153740. doi: 10.1371/journal.pone.0153740 (PMC4864230; doi:10.1371/journal.pone.0153740)
Supplement: S4 Table — (DOCX) [file pone.0153740.s011.docx]

**S4 Table. Published data on placebo arms in psoriasis randomised controlled studies**^1^

|  | # of patients^2^ |
| --- | --- |
| Average PASI75 | 4.5% |
| Range | 3.7 - 5.0% |
| Average # on placebo | 305 |
| Total # on placebo | 1524 |

^1^Data shown as reported from five randomised controlled studies listed in table 4.

^2^Shown are the number of patients reported in each study that were included on the placebo - only arm.
